# THE CO-EVOLUTION OF LONELINESS AND COMPANIONSHIPS IN AN ASSISTED LIVING COMMUNITY: A SOCIAL NETWORK ANALYSIS

**DOI:** 10.1093/geroni/igae098.0105

**Published:** 2024-12-31

**Authors:** Rebecca Mauldin, Jaclyn Kirsh, Kayo Fujimoto

**Affiliations:** University of Texas at Arlington, Arlington, Texas, United States; The University of Texas Arlington, Arlington, Texas, United States; The University of Texas Health Science Center Houston, Houston, Texas, United States

## Abstract

Loneliness and social isolation are distinct constructs that tend to be moderately correlated. The Evolutionary Theory of Loneliness posits that loneliness can motivate people to increase or improve their social ties. However, interrelated factors including loneliness, desired relationship quality/quantity, actual relationships, and appraisals of existing social networks may affect whether loneliness leads to increased social ties. In assisted living communities (ALCs) most opportunities for socializing with non-family members occur within the community, restricting residents’ pool of potential friends and companions. This study investigates the co-evolution of loneliness and companionship ties among older residents in an ALC using three waves of secondary data from the Social Experiences in Assisted Living (SEAL) study. We estimated a stochastic actor-oriented network-behavioral co-evolution model for companionship and loneliness dynamics in a network of 38 ALC residents to understand the reciprocal effects of loneliness and companionships. We found that increased levels of loneliness were significantly associated with a decreased likelihood of subsequently maintaining or adding companionship ties among other residents (ϴ = -.131, p <.01). Changes in companionship ties were not significantly associated with changes in levels of loneliness. Greater frequency of contact with family members was significantly negatively associated with the likelihood of companionship ties with other residents (ϴ = -.113, p <.05), but was not significantly associated with levels of loneliness. These findings do not support the hypothesis that increased levels of loneliness lead to maintaining or adding companions in the ALC and have implication for both theory and practice.

